# The m^6^A RNA Demethylase ALKBH9B Plays a Critical Role for Vascular Movement of Alfalfa Mosaic Virus in Arabidopsis

**DOI:** 10.3389/fmicb.2021.745576

**Published:** 2021-10-04

**Authors:** Mireya Martínez-Pérez, Concepción Gómez-Mena, Luis Alvarado-Marchena, Riad Nadi, José Luis Micol, Vicente Pallas, Frederic Aparicio

**Affiliations:** ^1^Instituto de Biología Molecular y Celular de Plantas, Consejo Superior de Investigaciones Científicas-Universitat Politècnica de Valencia, Avda, Valencia, Spain; ^2^Instituto de Bioingeniería, Universidad Miguel Hernández, Elche, Spain; ^3^Departamento de Biotecnología, Escuela Técnica Superior de Ingeniería Agronómica y del Medio Natural, Universitat Politècnica de Valencia, Valencia, Spain

**Keywords:** AMV, m^6^A, ALKBH9B, systemic infection, phloem transport

## Abstract

The *N*^6^-methyladenosine (m^6^A) pathway has been widely described as a viral regulatory mechanism in animals. We previously reported that the capsid protein (CP) of alfalfa mosaic virus (AMV) interacts with the Arabidopsis m^6^A demethylase ALKBH9B regulating m^6^A abundance on viral RNAs (vRNAs) and systemic invasion of floral stems. Here, we analyze the involvement of other ALKBH9 proteins in AMV infection and we carry out a detailed evaluation of the infection restraint observed in *alkbh9b* mutant plants. Thus, *via* viral titer quantification experiments and *in situ* hybridization assays, we define the viral cycle steps that are altered by the absence of the m^6^A demethylase ALKBH9B in Arabidopsis. We found that ALKBH9A and ALKBH9C do not regulate the AMV cycle, so ALKBH9B activity seems to be highly specific. We also define that not only systemic movement is affected by the absence of the demethylase, but also early stages of viral infection. Moreover, our findings suggest that viral upload into the phloem could be blocked in *alkbh9b* plants. Overall, our results point to ALKBH9B as a possible new component of phloem transport, at least for AMV, and as a potential target to obtain virus resistance crops.

## Introduction

*N*^6^-methyladenosine (m^6^A) is one of the most abundant modified bases included in the mRNAs of eukaryotes. It has been found in distinct types of RNAs and in the genomes of some viruses (Martínez-Pérez et al., 2017; Liang et al., 2018; Dang et al., 2019; Arribas-Hernández and Brodersen, 2020). In plants, this chemical modification is conducted by a methylation complex composed of *N*^6^-ADENOSINE-METHYLTRANSFERASE MT-A70-like (MTA, AT4G10760) and *N*^6^-ADENOSINE-METHYLTRANSFERASE NON-CATALYTIC SUBUNIT MTB (MTB, AT4G09980) and several cofactors, such as FKBP12-INTERACTING PROTEINS OF 30 KDA (FIP37, AT3G54170), PROTEIN VIRILIZER HOMOLOG (VIRILIZER, AT3G05680), and E3 UBIQUITIN-PROTEIN LIGASE HAKAI HOMOLOG (HAKAI, AT5G01160; Zhong et al., 2008; Shen et al., 2016; Růžička et al., 2017). Moreover, proteins characterized to remove this methyl group belong to the AlkB family of Fe(II)- and α-ketoglutarate-dependent dioxygenases (Van den Born et al., 2009; Nadi et al., 2018). In Arabidopsis, the AlkB family is composed of 14 proteins, but the demethylation activity has only been demonstrated experimentally for RNA DEMETHYLASE ALKBH9B (ALKBH9B, AT2G17970) and ALKBH10B (ALKBH10B, AT4G02940; Kawai et al., 2014; Duan et al., 2017; Martínez-Pérez et al., 2017). On the other hand, EVOLUTIONARILY CONSERVED C-TERMINAL REGIONS (ECT) 2, ECT3, and ECT4 (AT3G13460, AT5G61020, and AT1G55500, respectively) have been described as proteins that recognize and process m^6^A-modified RNAs in Arabidopsis (Arribas-Hernández et al., 2018; Scutenaire et al., 2018; Wei et al., 2018). Diverse studies point to m^6^A as a new post-transcriptional pathway to modulate viral infections (Williams et al., 2019). There are multiple examples of such modulation in animals, but not in plants.

Alfalfa mosaic virus (AMV) belongs to the *Alfamovirus* genus of the *Bromoviridae* family, and its genome consists of three molecules of positive single-strand RNAs (Bujarski et al., 2019). RNA 1 and RNA 2 encode the two subunits of the viral replicase (P1 and P2), respectively. RNA 3 is bicistronic and encodes the movement protein (MP) and, through a subgenomic RNA (RNA 4), the capsid protein (CP; Bol, 2008). Viral RNAs (vRNAs) harbor a cap-like structure at the RNA 5'-terminus and lack polyadenylation at the 3'-terminus. Instead, the 3' untranslated region (UTR) of AMV and ilarviruses present homologous sequences that can form two mutually exclusive conformations: one predicted to fold into linear arrays of several hairpin structures, and a second one resembling the tRNA-like structure (TLS) described for bromo- and cucumoviruses (Pallas et al., 2013). This conformation change seems to act as a molecular switch from translation to replication (Aparicio et al., 2003; Chen and Olsthoorn, 2010). On the other hand, the mechanism used for AMV transport to adjacent cells through plasmodesmata (PD) remains unclear, although several observations point to a tubule-guided mechanism that would not require virion assembly (Van Der Vossen et al., 1994; Kasteel et al., 1997; Zheng et al., 1997; Sánchez-Navarro and Bol, 2001). Besides, to systemically invade the whole plant, viruses need to reach the vascular system through a highly regulated process (Pallas and García, 2011; Navarro et al., 2019). To reach the sieve elements (SE), viruses have to overcome diverse cellular barriers: bundle sheath (BS), vascular parenchyma (VP), and companion cells (CC; Hipper et al., 2013; Navarro et al., 2019). Recent studies have shown that viral infections cause substantial transcriptional and translational reprogramming of phloem expressing genes both to interfere with host defense mechanisms and to promote their own systemic transport (Kappagantu et al., 2020). To accomplish long-distance transmission, AMV requires a functional CP to form viral particles (Tenllado and Bol, 2000; Herranz et al., 2012). Nonetheless, the exact molecular mechanism involved in phloem load and unload of AMV remains poorly understood so far.

In previous work, we found that the AMV CP interacts with ALKBH9B and we showed that this protein decreases m^6^A relative abundance along AMV vRNAs. In turn, this promotes viral accumulation in Arabidopsis since the virus showed an impaired systemic invasion capability in *alkbh9b* plants (Martínez-Pérez et al., 2017; Alvarado-Marchena et al., 2021). Here, we discard that the other ALKBH9 close paralogous proteins affect AMV infection and we make a deep analysis of the infection restriction observed in *alkbh9b* plants. Furthermore, *via* northern blots, tissue printings, and *in situ* hybridization assays from several tissues and at different times, we have determined which viral cycle steps or cell boundaries are affected by the absence of ALKBH9B in Arabidopsis plants.

## Materials and Methods

### Microscopy and Morphometry

All Arabidopsis thaliana (L.) Heynh. plants studied in this work were homozygous for the mutations indicated in each case. The Nottingham Arabidopsis Stock Center provided seeds of the Col-0 accession (N1092; referred to herein as WT), as well as of the *alkbh9a* (N693727), *alkbh9b* (N515591) and *alkbh9c* (N521775) mutants. For rosette morphology analyses and to obtain multiple mutant genetic combinations, plants were grown under sterile conditions on 150-mm diameter Petri dishes, containing 100ml half-strength Murashige and Skoog agar medium, 0.6% Gelrite Agar (Duchefa), and 1% sucrose, at 20°C±1°C, 60–70% relative humidity, and continuous illumination of ≈ 75μmol·m^−2^·s^−1^, and transferred into pots 21days after stratification (das) as previously described (Ponce et al., 1998). Sixteen evenly spaced seeds were sown per plate. Crosses were performed as previously described (Quesada et al., 2000). Photographs of rosettes were taken on a Nikon SMZ1500 stereomicroscope equipped with a Nikon DXM1200F digital camera. Rosette leaf area was calculated using Phenovein (version r214) of the MeVisLab software (version 3.1.1; Bühler et al., 2015).

### Genotyping and Semiquantitative Gene Expression Analysis

The presence and position of annotated T-DNA insertions were confirmed by PCR amplification using the LbB1.3 primer and gene-specific primers (Supplementary Table 1). For RT-PCR, total RNA was extracted from seedlings collected 15 das using TRIzol (Invitrogen, Thermo Fisher Scientific) and contaminating DNA was removed using a TURBO DNA-free Kit (Invitrogen). First-strand cDNA was synthesized using random hexamers and the Maxima Reverse Transcriptase system (Fermentas). The house-keeping *ORNITHINE TRANSCARBAMYLASE* (*OTC*) gene was used as an internal control for relative expression analysis, as previously described (Quesada et al., 1999). Three different biological replicates were used. Sequence data can be found at The Arabidopsis Information Resource^1^ under the following accession numbers: *ALKBH9A* (At1g48980), *ALKBH9B* (At2g17970), *ALKBH9C* (At4g36090), and *OTC* (At1g75330).

### Plant Growth Conditions, Virus Inoculation, and Northern Blots

Nicotiana benthamiana and Arabidopsis thaliana plants were grown in 6-cm diameter pots in a growth chamber with a photoperiod of 25°C-16-h light/20°C-8-h dark. Plants were mechanically inoculated with purified virions (1mg/ml) of AMV PV0196 isolate (Plant Virus Collection, DSMZ) in 15mm sodium phosphate buffer pH 7. Detection of vRNAs was carried out by northern or direct-dot blot analysis. Inoculated leaves, non-inoculated rosette leaves, upper systemic floral stems, and roots were grounded in liquid nitrogen with mortar and pestle, and total RNA was extracted from 0.1g leaf material using EXTRAzol reagent protocol (Blirt). 0,5μg of total RNA was denatured by formaldehyde treatment and analyzed by northern blot. Otherwise, for direct-dot blot analysis, 3μl of total RNA was directly blotted onto nylon membranes. vRNAs were visualized on blots using digoxigenin-labeled riboprobes to detect the four vRNAs of AMV. Synthesis of the digoxigenin-labeled riboprobes, hybridization, and digoxigenin detection procedures was carried out as previously described (Pallás et al., 1998).

### Tissue Printing of Inoculated Leaves and Floral Stems

Leaves of 3-week-old WT and *alkbh9b* plants were inoculated with AMV virion and directly printed onto a nylon membrane at 4 dpi by pressing the lower side as previously described (Más and Pallás, 1995). To analyze AMV systemic invasion in all *alkbh9* mutant combinations, floral stems were cut at 12 dpi and directly pressed onto the nylon membranes. Hybridization procedures were carried out as previously described (Pallás et al., 1998).

### *In situ* Hybridization Assays

RNA *in situ* hybridization using a digoxigenin-labeled probe was performed on 8μm longitudinal paraffin sections as described previously (Gómez-Mena and Roque, 2018). Plant material was fixed in a solution containing 4% paraformaldehyde and 2.5% glutaraldehyde in 0.1M phosphate buffer (pH 7.2) for 4h at 4°C and embedded in paraffin. Hybridization was performed at 55°C overnight. After hybridization and immunodetection, slides were examined and photographed with a Leica DM 5000 equipped with a digital camera. When required, several images were stitched together using the stitching function of Leica Application Suit v4.9.

### Virion Stability Assays

The RNase sensitivity assay was performed according to Gao et al. (2021). For this, 0.1g of systemic tissue previously infected with AMV was ground in liquid nitrogen and was incubated in 100μl of PIPES (CAS:5625-37-6) buffer (50mm PIPES, pH 6.7, 0.1% Tween 20) at 37°C. Total RNA was extracted at different time points and was analyzed by northern blot.

### Protoplast Isolation and Inoculation

Isolation and inoculation of WT and *alkbh9b* protoplasts were carried out as previously described (Yoo et al., 2007) with some modifications. Well-expanded leaves from 3-week-old plants were chosen, and their undersides were slightly rubbed with carborundum. The leaves were incubated on the enzyme solution [20mM MES (pH 5.7), 1.5% (wt/vol) cellulase R10, 0.4% (wt/vol) macerozyme R10, 0.4 M mannitol, 20mm KCl, 10mM CaCl_2_, 1–5mM β-mercaptoethanol, and 0.1% BSA] for 3h at room temperature. The enzyme/protoplast solution was diluted with an equal volume of W5 solution [2mM MES (pH 5.7), 154mM NaCl, 125mM CaCl_2_, and 5mM KCl] before filtration with miracloth. After that, a sucrose cushion was performed adding approximately 1ml of 20% sucrose to the bottom of the tube with a Pasteur pipette and centrifugated at 200g for 5min. Protoplasts from the interphase were recovered and diluted in 1 volume of W5 and centrifuged at 200g for 3min. After removing the supernatant, the protoplasts were resuspended in 1ml in W5 solution and kept on ice for 30min. The supernatant was removed, and the protoplasts were resuspended in MMG solution [4mM MES (pH 5.7), 0.4 M mannitol, and 15mM MgCl_2_]. For the transfection process, 10μl of vRNA (10–20μg extracted from AMV virions), 200μl of protoplasts (5×10^5^ cells), and 210μl of PEG solution [40% (wt/vol) PEG4000, 0.2 M mannitol, and 100mM CaCl_2_] were gently mixed in a 2-ml microfuge tube and incubated at room temperature for 7min. To stop the transfection reaction, the samples were diluted with 840μlW5 solution at room temperature and centrifuged at 100g for 2min. After removing the supernatant, protoplasts were resuspended with 1ml WI [4mM MES (pH 5.7), 0.5 M mannitol, and 20mM KCl] and incubated at room temperature for 18h. Total RNA extraction with EXTRAzol reagent protocol (Blirt) was carried out to analyze viral accumulation by northern blot analysis.

### Digoxigenin-Labeled Probes

A region of 200 nucleotides from the AMV RNA1 and RNA2 was amplified with specific primers of which the antisense primer contained the T7 RNA polymerase promoter. Amplified cDNA products and a plasmid containing a fragment of the ORF AMV CP linearized with the appropriate restriction enzyme were used as templates to transcribe RNA digoxigenin-labeled probes using T7 RNA polymerase and following manufacturer’s recommendations (Takara Bio Inc. Shiga, Japan). A mixture of Dig RNA 1, Dig RNA 2, and Dig-CP (100ng/each) or only the Dig-CP (500ng) was used as a probe for northern blot and *in situ* hybridization assays, respectively.

## Results

### ALKBH9B Is the Only ALKBH9 Involved in AMV Infection

**Figure 1:**
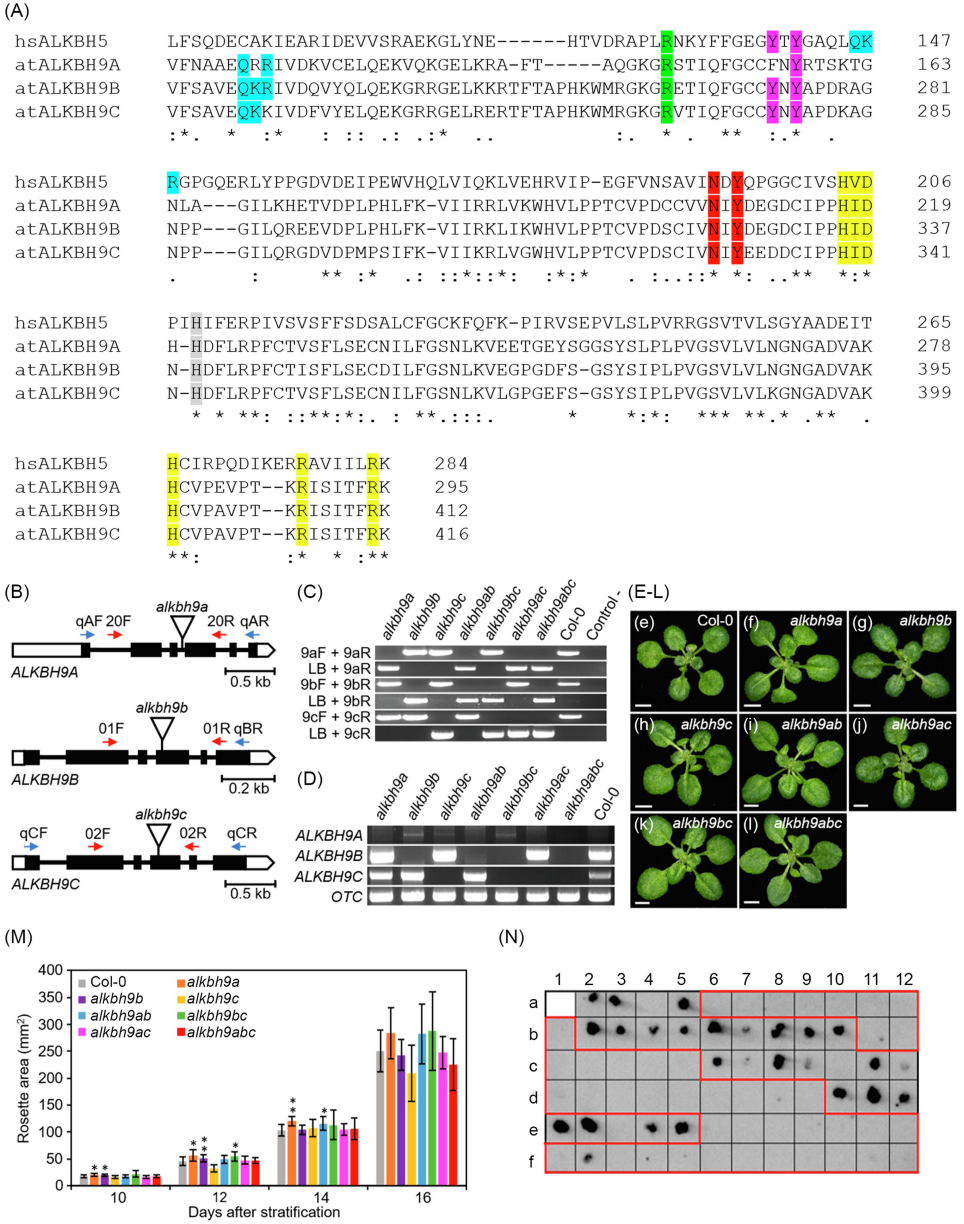
ALKBH9A and ALKBH9C are not involved in AMV systemic infection. **(A)** Multiple alignment of the amino acid sequences of the AlkB domain from ALKBH9 Arabidopsis paralogs and human ALKBH5. Protein sequences were obtained from the Arabidopsis Information Resource (https://www.arabidopsis.org/). The alignment was obtained with Clustal Omega 1.2.4 with default settings. Asterisks and periods in the consensus line indicate identical and similar residues, respectively. Numbers indicate residue positions. Residues/motifs involved in binding or interactions stabilization in hsALKB5 are highlighted in different colors (see main text). at, *Arabidopsis thaliana*; hs, *Homo sapiens*. **(B)** Schematic representation of the structure of the *ALKBH9A*, *ALKBH9B*, and *ALKBH9C* genes, with indication of the positions of the T-DNA insertions carried by the *alkbh9a*, *alkbh9b*, and *alkbh9c* mutants. Boxes and lines between boxes represent exons and introns, respectively. Open and black boxes represent untranslated and coding regions, respectively. Triangles represent T-DNA insertions. The oligonucleotides (not drawn to scale) used as RT-PCR and PCR primers are represented as horizontal blue and red arrows, respectively. The following abbreviations are used for oligonucleotide names throughout this figure: LB for LbB1.3; 20F/R for SALK_204823C_F/R; 01F/R for SALK_015591_F/R; 02F/R for SALK_021775_F/R; Lb for LbB1.3a; 9aF/R for qALKBH9a_F/R; 9bF/R for qALKBH9b_Rb; and 9cF/R for qALKBH9c_F/R (see Supplementary Figure Legends). **(C)** Genotyping of the insertional single and multiple *alkbh9* mutants studied in this work. The presence of the T-DNA insertions shown in **(B)** was visualized by agarose gel electrophoresis of the PCR amplification products obtained using the primer pairs indicated, one of which is specific to the T-DNA (LB in the figure) and the remaining ones of the disrupted genes. For simplicity, the following non-standard abbreviations are used for genotypes throughout this figure: *alkbh9ab* for the *alkbh9a alkbh9b* double mutant; *alkbh9ac* for *alkbh9a alkbh9c*; *alkbh9bc* for *alkbh9b alkbh9c*; and *alkbh9abc* for the *alkbh9a alkbh9b alkbh9c* triple mutant. **(D)** Visualization by agarose gel electrophoresis of the semiquantitative RT-PCR amplification products obtained from *ALKBH9A*, *ALKBH9B*, *ALKBH9C*, and *OTC* mRNAs. RNA was extracted from plants of the genotypes indicated, some of which are abbreviated as mentioned above. The primers used are also abbreviated as mentioned above. **(E**–**L)** Rosettes of (e) the Col-0 WT, the (f) *alkbh9a*, (g) *alkbh9b*, and (h) *alkbh9c* single mutants, the (i) *alkbh9a alkbh9b*, (j) *alkbh9a alkbh9c*, and (k) *alkbh9b alkbh9c* double mutants, and (l) the *alkbh9a alkbh9b alkbh9c* triple mutant. Photographs were taken 14days after stratification (das). Scale bars indicate 2mm. **(M)** Rosette growth progression of the *alkbh9* single and multiple mutants studied in this work. Rosette area was measured for the genotypes indicated at 10, 12, 14, and 16days after stratification (das). Twelve rosettes were measured per genotype. Asterisks indicate values significantly different from WT (Col-0) in a Student *t* test (*n*=12; ^*^p<0.05, ^**^p<0.001). Error bars indicate standard deviation. **(N)** Tissue printing of floral stems from infected plants: WT (a2-a5), *alkbh9b* (a6-b1 and b11-c5), *alkbh9a* (b2-b10), *alkbh9c* (c6-c12), *alkbh9ab* (d1-d9), *alkbh9ac* (d10-e5), *alkbh9bc* (e6-f3), and *alkbh9abc* (f4-f12) plants.

In Arabidopsis, ALKBH9 members form a specific clade including *ALKBH9A*, *ALKBH9B*, and *ALKBH9C* genes (Mielecki et al., 2012; Martínez-Pérez et al., 2017). Using the online tool Clustal Omega, we obtained the alignment between the amino acid sequences of the AlkB domain (residues 222 to 412 in ALKBH9B) from these three proteins and human ALKBH5 (hsALKBH5, residues 85 to 284; Figure 1A). Remarkably, the AlkB domain and some amino acids described as key residues for the m^6^A demethylation activity of hsALKBH5 (highlighted in Figure 1A) are quite conserved in the three ALKBH9 Arabidopsis co-orthologues (Feng et al., 2014; Lu et al., 2014; Alvarado-Marchena et al., 2021). Thus, we observed that the known HxD/E·· H and RxxxxxR motifs are conserved between the three proteins (Figure 1A, highlighted in yellow). According to previous authors, while the HxD/E···H motif and the first arginine (R) of RxxxxxR are involved in the binding to iron [Fe (II)] and 2-oxoglutarate (2-OG), the second R (R411 in ALKBH9B) seems to be related to the substrate specificity of AlkB (Bratlie and Drabløs, 2005; Lu et al., 2014). Moreover, the N and Y residues involved in 2-OG stabilization are conserved in all ALKBH9 paralogues (Figure 1A, highlighted in red, N324 and Y326 in ALKBH9B), whereas only one of the Y residues directly binding m^6^A is conserved in ALKBH9A (Figure 1A, Y273 and Y275 in ALKBH9B, in fuchsia). Likewise, instead of the characteristic hsALKBH5 motif QKR (position 170 in ALKBH9B), which is probably involved in the binding to the substrate, ALKBH9A and ALKBH9C present QRR and QKK, respectively (Figure 1A, in blue). On the other hand, an arginine residue essential for hsALKBH5 activity (Feng et al., 2014) is also conserved between the three ALKBH9 proteins (Figure 1A, R265 in ALKBH9B, highlighted in green). Finally, a histidine residue specifically found in hsALKBH5 but not in the rest of AlkB is also present in all ALKBH9 proteins (Figure 1A, H339 in ALKBH9B, highlighted in gray). Overall, this hints at m^6^A demethylation activity of ALKBH9A and ALKBH9C that also could affect AMV propagation. Therefore, we carried out a study to check whether ALKBH9A and/or ALKBH9C might be also playing a role in AMV systemic infection.

Thus, we intercrossed the *alkbh9a* (SALK_204823C), *alkbh9b* (SALK_015591), and *alkbh9c* (SALK_021775) insertional single mutants (Figure 1B), obtaining all possible multiple genetic combinations: the *alkbh9a alkbh9b, alkbh9a alkbh9c* and *alkbh9b alkbh9c* double mutants, and the *alkbh9a alkbh9b alkbh9c* triple mutant, to which we will refer here, for simplicity, as *alkbh9ab alkbh9ac, alkbh9bc*, and *alkbh9abc*, respectively. We confirmed by PCR the presence of the corresponding T-DNA insertions in homozygosis in all single and multiple mutants (Figure 1C). We also confirmed by semiquantitative RT-PCR the absence of full-length transcripts of the disrupted genes (Figure 1D, Supplementary Figure Legends). As expected, we found that *ALKBH9A* expression was not detected in whole seedlings of the Col-0 wild type (referred to herein as WT); unexpectedly, however, it was very low, but detectable in the *alkbh9b* and *alkbh9bc* mutants and to a lesser extent in *alkbh9c* (Figure 1D; Duan et al., 2017). A compensation mechanism like this has already been observed among other paralog groups in Arabidopsis; examples are the paralog gene pairs encoding the pathogen responsive VESICLE-ASSOCIATED MEMBRANE PROTEINS721 (Kwon et al., 2008; El-Brolosy and Stainier, 2017) and VAMP722 or the nucleolar proteins NUCLEOLIN1 (a ribosome biogenesis factor) and NUC2 (Durut et al., 2014). We studied the morphological phenotype at the level of whole rosette growth progression in *alkbh9* single and multiple mutant seedlings. Overall, rosettes of the mutant plants studied were barely distinguishable from WT (Figures 1E–M).

The effect on viral systemic movement of the *alkbh9a*, *alkbh9b*, and *alkbh9c* single mutants, the *alkbh9ab*, *alkbh9ac*, and *alkbh9bc* double mutants, and the *alkbh9abc* triple mutant was analyzed by detecting AMV accumulation in floral stems by tissue printing at 12days post-inoculation (dpi). As shown in Figure 1N, the viral systemic movement was impaired only in plants lacking ALKBH9B (highlighted in red), indicating that neither ALKBH9A nor ALKBH9C is required for the systemic invasion of the plant. This result highlights the specificity of the interaction between AMV infection and ALKBH9B.

### ALKBH9B Might Be Required by AMV to Invade the Vascular Tissues

**Figure 2:**
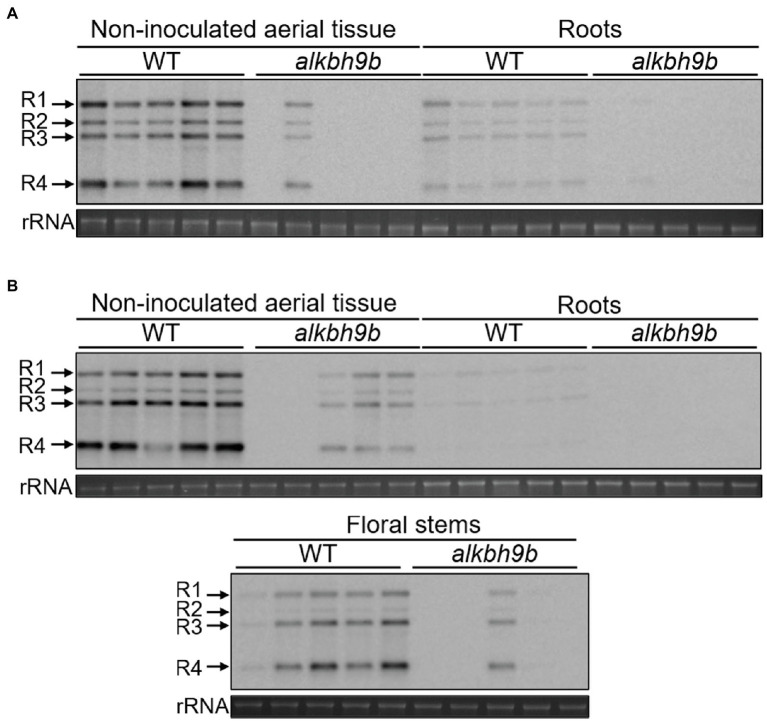
AMV systemic infection of diverse tissues is severely impaired in *alkbh9b* plants. Representative northern blots of different tissues (indicated on top of each panel) from five WT and *alkbh9b* plants at 11 **(A)** and 15 **(B)** dpi. Five biological replicates of each tissue are shown. Positions of the vRNAs are indicated on the left. Ethidium bromide staining of ribosomal RNAs (rRNA) was used as RNA loading control.

The results shown in Figure 1N are in line with our previous work reporting that AMV succeeded in infecting only 9% of the floral stems of mutant plants at 14 dpi, whereas that percentage increased until 90–100% in WT plants (Martínez-Pérez et al., 2017). However, the evaluation of AMV infection in total non-inoculated aerial tissue by dot blot revealed that 40% of *alkbh9b* plants presented a systemic infection compared to 93% of WT plants at 20 dpi (Supplementary Figure 1). This inconsistency encouraged us to analyze more precisely the evolution of the systemic infection in plants. Thus, we compared the viral titers in different non-inoculated tissues (roots, rosette leaves, and floral stems). After total RNA extraction, vRNAs load in these tissues was evaluated by northern blot at 11 and 15 dpi (Figures 2A,B, respectively). Although the lower susceptibility of *alkbh9b* plants to AMV was evident in all cases, it was especially notable in roots and floral stems, in which we practically observed a block of the viral infection. Considering that to invade upper sink leaves and roots, viruses enter the phloem of the inoculated leaves (Hipper et al., 2013; Navarro et al., 2019), our results suggest that AMV phloem loading in *alkbh9b* plants is restricted.

**Figure 3:**
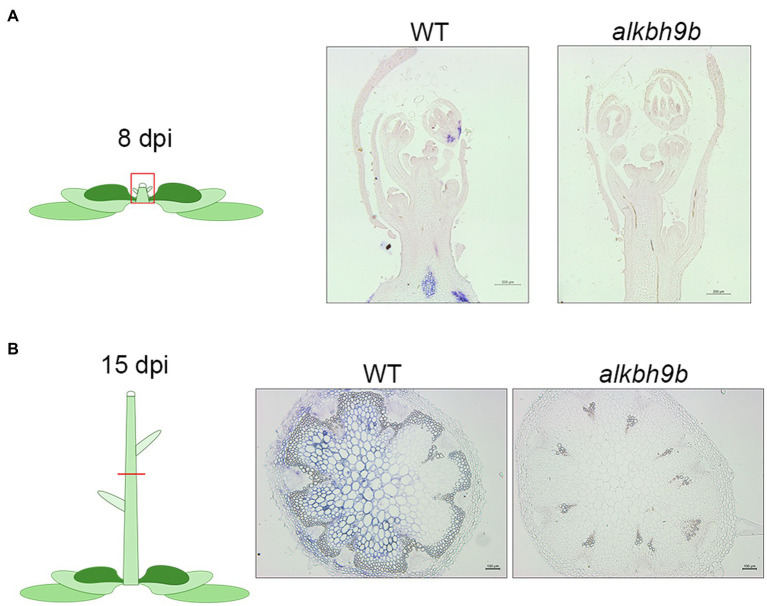
AMV usually does not invade vascular tissue in AMV plants. Representative pictures of the SAMs **(A)** and floral stems **(B)** from WT and *alkbh9b* plants at 8 and 15 dpi, respectively. Scale bars correspond to 200μm (a) or 100μm (b).

Next, we evaluated in more detail the viral progression in the shoot apical meristems (SAM) and floral stems at 8 and 15 dpi, respectively, detecting AMV RNA4 by *in situ* hybridization (Figure 3, Supplementary Figure 3). At 8 dpi, no hybridization signal was observed in the SAM of WT plants, as expected accordingly to the general knowledge that viruses cannot invade the SAM (Gosalvez-Bernal et al., 2006; Wu et al., 2020). However, we could observe the presence of vRNAs patches in both developing flowers and floral stems. No signal was observed in *alkbh9b* plants in these same tissues (Figure 3A, Supplementary Figure 2). Furthermore, at 15 dpi, AMV RNA was still not detected in transverse sections of the inflorescence stems from *alkbh9b* plants, whereas the hybridization signal covered almost completely those from WT plants (Figure 3B). Overall, these observations show that AMV systemic infection is generally impaired by the absence of ALKBH9B and viral RNA is unable to reach floral stems and roots. More remarkably, these new findings suggest that ALKBH9B is involved, somehow, in the viral upload to the phloem in Arabidopsis, and thus, when this protein is not present, AMV very rarely invades the vascular tissues.

In contrast to cell-to-cell movement, as explained above, AMV needs to form viral particles to move systemically (Sánchez-Navarro et al., 2006). The diminished systemic invasion observed in *alkbh9b* plants could be caused by compromised stability of the AMV particles. To check this hypothesis, we evaluated the stability of viral particles obtained from WT and *alkbh9b* plants. It would be expected that encapsulated vRNAs were hardly processed by RNases. Hence, WT- and *alkbh9b*-infected leaves were homogenized with PIPES buffer and, after different times of incubation at 37°C, vRNAs levels were evaluated by northern blot assays (Supplementary Figure 3). While ribosomal RNAs were completely degraded after 30min of incubation, vRNAs from both WT and mutant plants were intact to the same extent. Therefore, the absence of ALKBH9B activity does not affect the stability of AMV viral particles.

### AMV Cell-to-Cell Movement Among Mesophyll Cells Is Reduced in the Absence of ALKBH9B

**Figure 4:**
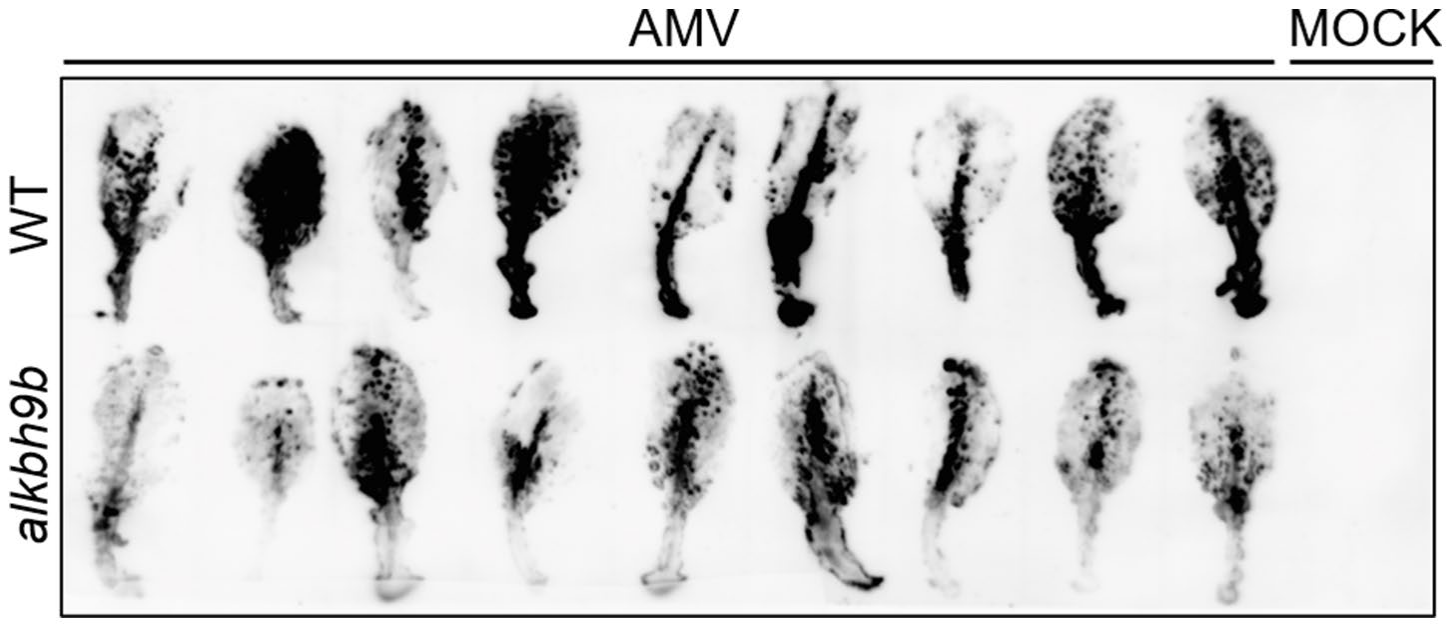
AMV infection is impaired in inoculated leaves of *alkbh9b* plants. Tissue printing of inoculated leaves from WT and *alkbh9b* plants at 4 dpi. Nine biological replicates of each genotype are shown.

**Figure 5:**
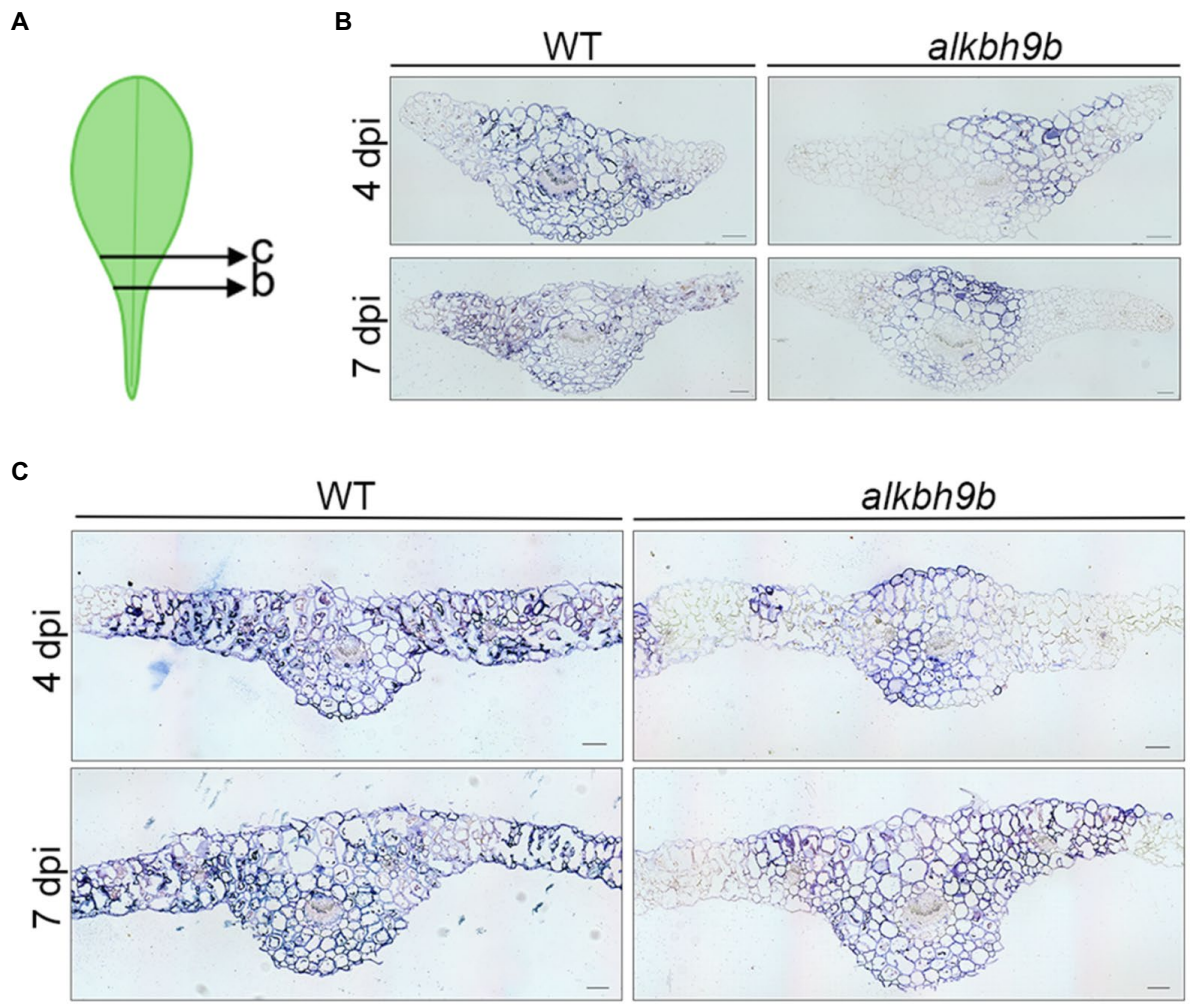
AMV fails to invade the vascular tissue of *alkbh9b* plants. **(A)** Picture showing the approximate sections used for *in situ* hybridization assay. **(B,C)** *In situ* hybridization of the petioles **(B)** and blades **(C)** of AMV-inoculated leaves at 4 and 7 dpi (indicated in each panel) from WT and *alkbh9b* plants. Scale bars correspond to 100μm.

We further evaluate both AMV cell-to-cell movement among mesophyll cells by analyzing the viral distribution in the inoculated leaves. Inoculated leaves from WT and *alkbh9b* plants were directly printed onto nylon membranes, and AMV RNAs were detected by hybridization with dig-AMV probe (Figure 4). While most leaf surfaces were already infected in WT plants at 4 dpi, the infection in *alkbh9b* leaves did not reach the petioles yet. Since this may indicate a delay in the viral infection progress, we addressed *in situ* hybridization experiments of sections of leaf petioles and leaf blades of inoculated leaves using a dig-RNA4 probe to detect AMV RNA 3 and sgRNA 4 (Figure 5A). In WT petioles, the hybridization signal covered most of the tissue already at 4 dpi, whereas *alkbh9b* mutant petioles showed small patches even at 7 dpi (Figure 5B). Moreover, when the analysis was extended to leaf blades, we found that, while AMV signal was observed all over the section in WT leaf blades, only individual patches of hybridization were seen in *alkbh9b*, pointing to a less efficient invasion of mesophyll cells in the inoculated leaves when this protein is not present (Figure 5C, Supplementary Figure 4). Remarkably, the lack of infection in vascular tissues from *alkbh9b* even when the infection has reached the directly surrounding cells of this area would explain the failure of the virus to invade the floral stems (see Figure 3, Supplementary Figure 2).

### The Absence of ALKBH9B Affects the Viral Replication Cycle

**Figure 6:**
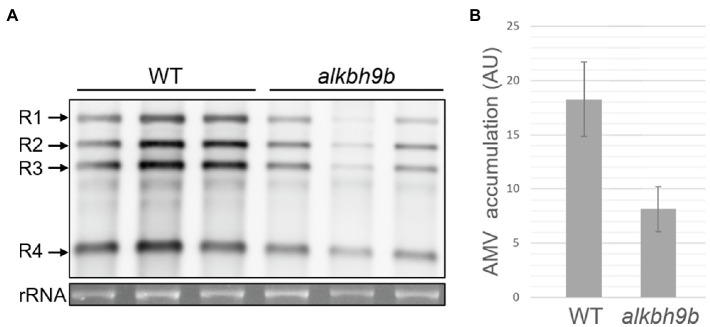
AMV replication cycle is affected in *alkbh9b* protoplasts. **(A)** Representative northern blot of AMV-inoculated protoplasts isolated from WT and *alkbh9b* plants. Three biological replicates of each genotype are shown. Positions of the AMV RNAs are indicated on the left. Ethidium bromide staining of rRNA was used as RNA loading control. **(B)** Graphic showing the quantification of panel A. Error bars indicate standard deviation.

The phenotypic defect in *alkbh9b* plants consisting of a slower cell-to-cell movement may occur, at least partially, as a consequence of any defects in earlier stages of the viral cycle (replication, translation or RNA stability). In fact, *in situ* hybridization assays did not only display a less widespread infection, but also a weaker viral signal in *alkbh9b* plants (Figure 5, Supplementary Figure 4). To properly check this hypothesis, we inoculated isolated protoplasts from WT and *alkbh9b* plants with purified AMV vRNAs and compared the infection levels by northern blot analysis. The result showed that viral load was lower in protoplasts coming from mutant plants (Figure 6), suggesting that ALKBH9B is involved at least in one of these stages: viral replication, translation, or vRNAs stability.

## Discussion

We previously reported that AMV infection was severely impaired at local level and almost blocked in *alkbh9b* floral stems compared to WT plants. We proposed that the binding between the CP of AMV and the m^6^A demethylase protein ALKBH9B leads to the decrease in m^6^A/A ratio in the vRNAs and, consequently, promotes viral infection (Martínez-Pérez et al., 2017). Here, we have evaluated the stages of the viral infection cycle potentially affected by the deregulation of the vRNAs-m^6^A methylation in *alkbh9b* plants. We first assessed, however, the potential effects that the other two ALKBH9 proteins could have in the AMV infection cycle. Even though some essential amino acids for m^6^A demethylation activity are conserved in these proteins, we found that ALKBH9A and ALKBH9C are not involved in the systemic movement of this virus. This result highlights the specificity of the AMV-ALKBH9B interaction, which could be related to either the expression pattern and/or the subcellular localization of ALKBH9 proteins. ALKBH9C is mainly expressed in leaves, but between 70% and 90% of the protein was found to accumulate in the nucleus, whereas ALKBH9A is virtually undetectable (Mielecki et al., 2012; Duan et al., 2017). In contrast, ALKBH9B is one of the most expressed Arabidopsis ALKB genes, mainly in the apical meristem and buds (Duan et al., 2017), and localizes exclusively in the cytoplasm, where AMV replication takes place (Ibrahim et al., 2012). However, although there is no evidence so far, we cannot rule out the possibility that neither ALKBH9A nor ALKBH9C can remove methyl groups from m^6^A-modified RNAs.

The partial resistance to AMV observed in *alkbh9b* plants is extremely remarkable. The detailed analysis of the AMV invasion of the plant included in this work reveals that transport to the upper non-inoculated leaves and, especially, to the roots is severely impaired in *alkbh9b* plants. This observation, together with the *in situ* hybridization analyses that revealed a clear lack of hybridization signal in the phloem of *alkbh9b* floral stems, suggests that ALKBH9B might modulate the upload of AMV into the phloem.

First, we reasoned that, as infection levels are lower in inoculated leaves of *alkbh9b* compared to WT plants, cell-to-cell movement among mesophyll cells and/or earlier infection stages must be also affected in the mutant. Previously, we demonstrated that ALKBH9B forms discrete cytoplasmic granules corresponding to siRNA bodies and non-mediated decay granules suggesting that ALKBH9B activity might be related to mRNA decay processes (Martínez-Pérez et al., 2017). Thus, the decrease in the viral load observed in *alkbh9b* protoplasts might be caused by the detriment to stability, availability, abundance, and half-life produced by an excessive m^6^A methylation in the vRNAs. Interestingly, *in situ* hybridization assays of the inoculated leaves revealed the presence of individual small patches of hybridization pointing to possible limitations in cell-to-cell movement. It is conceivable that putative interactions between PD resident proteins and AMV RNAs – and, consequently, cell-to-cell movement – could be affected by the methylation status of the vRNAs. Reinforcing this hypothesis, Liu et al. (2017) found that m^6^A abundance increases the recognition of RNAs by the heterogeneous nuclear ribonucleoprotein G, which demonstrates that m^6^A deposition on RNAs can induce structural changes that alter the accessibility of the RNA to RNA-binding proteins. In the same line, the nuclear export of mRNAs from human immunodeficiency virus-1 (HIV-1) requires the binding of HIV-Rev protein to HIV-Rev Response Element RNA (RRE) and the m^6^A methylation of two conserved adenosine residues of the stem loop II region of RRE favors this interaction (Lichinchi et al., 2016).

After infection of the mesophyll cells and for long-distance movement, viruses have to successively cross, by cell-to-cell movement, the boundaries of BS, VP, and CC to finally enter into the SE. The CC-SE complex can be considered as the traffic control center of the phloem (Oparka and Turgeon, 1999), and it is a key regulatory checkpoint to restrict virus entry by avoiding systemic transport (Navarro et al., 2019). Diverse studies have reported that PD located in these boundaries contain specific host mechanism(s) that can prevent cell-to-cell transport of incompatible viruses and that, in some cases, are host and virus-specific (Vuorinen et al., 2011; Hipper et al., 2013; Navarro et al., 2019). For example, the Arabidopsis protein VIRUS SYSTEMIC MOVEMENT (VSM1) is specifically required for loading tobamoviruses into de SE, whereas systemic transport of the carmovirus tobacco crinkle virus is not affected in the *vsm1* mutant (Lartey et al., 1998). Moreover, it was identified a resistant cultivar of soybean in which cowpea chlorotic mottle bromovirus is blocked in BS cells, indicating the existence of a specific host machinery at the BS/VP boundary (Goodrick and Kuhn, 1991). Also, specific point mutations in the CP of tobacco mosaic virus (TMV) impede the viral transport through the VP/CC boundary in *Nicotiana tabacum* (Ding et al., 1996). Therefore, as proposed above for cell-to-cell movement, putative interactions between PD resident proteins of phloem-cellular components and AMV RNAs and/or virions may be affected by the m^6^A status of the vRNAs. Among other functions, methylation has been proposed to affect the systematic transport of plant RNAs modulating their stability (Wang et al., 2021) or the recognition of TLS by tRNA-specific proteins involved in viral transport (Lezzhov et al., 2019). Here, we have found that the lack of ALKBH9B does not affect the viral particle stability, but m^6^A might, for instance, affect the AMV TLS and, consequently, the host-virus interactions required for the transport among specific phloem cells.

On the other hand, phloem tissues do not remain invariable and unmodified under virus infection. Studies into alterations of the phloem-associated transcriptome and proteome induced by viruses showed that viral infections induce a massive regulation of phloem-expressed genes (Kappagantu et al., 2020). Therefore, phloem is important for the antiviral defense response and it is also a target of the host reprogramming induced by viruses. For example, more than 5,000 phloem-specific differentially expressed genes were identified in TMV-infected Arabidopsis plants (Collum and Culver, 2017). Moreover, several host factors that function either facilitating or restricting virus long-distance movement have been identified (Pallas et al., 2011; Hipper et al., 2013; Navarro et al., 2019). Hence, m^6^A methylation status of the phloem transcriptome in *alkbh9b* plants could somehow regulate viral load into the vascular tissue by altering the regulation of the PD permeability. However, the lack of any antiviral phenotype of *alkbh9b* plants against CMV (Martínez-Pérez et al., 2017) suggests that it might be a more specific mechanism for AMV. Other antiviral defense pathways, such as RNA silencing (Cordero et al., 2017) and recessive (Guiu-Aragonés et al., 2016) or dominant resistance (Chisholm et al., 2001), have been related to the inhibition of viral long-distance movement *via* the phloem (Vuorinen et al., 2011), and thus, m^6^A might constitute a new one. In this sense, Yang et al. (2019) described that 5-methylcytosine (m^5^C) is enriched in mobile mRNAs and found that the transport through the phloem of transcripts of two genes, TRANSLATIONALLY CONTROLLED TUMOR PROTEIN 1 and HEAT SHOCK COGNATE PROTEIN 70.1 (HSC70.1), was reduced in mutants deficient in m^5^C methylation. According to our results and contrary to m^5^C, increased levels of m^6^A would decline AMV loading into the phloem and consequently long-distance transport. It is worth noting that Wang et al. (2021) recently discussed that the reasons why m^5^C favors the systemic movement of RNAs are unknown so far, and they could be diverse. This would justify the discrepancies between the processing of RNAs harboring one or another modification.

Finally, the reduced accumulation levels of vRNAs in *alkbh9b* cells might also influence the viral systemic movement failure of AMV. In this line, a recent work demonstrated a link between the mRNA abundance and its long-distance mobility. The recognition of a sequence motif in the RNA by a PD-associated chaperone and its subsequent transport through PD would be favored when the mRNA concentration is close to reach the dissociation constant of that specific interaction (Calderwood et al., 2016). However, the block of the AMV systemic movement is difficult to explain by this hypothesis. A previous study on the infection capability of different AMV mutants showed that mutants with reduced replication rates in protoplasts were able to sustain almost WT-like systemic invasion. In contrast, other AMV mutants with replication levels similar to WT in protoplasts showed an impaired systemic movement (Tenllado and Bol, 2000).

An intriguing observation was that the percentage of infected non-inoculated rosette leaves was higher than that observed for floral stems in *alkbh9b* plants. This result could be explained by the existence of an alternative way to invade the plant from source to sink leaves that avoids the phloem and that has been demonstrated for a strain of potato virus A (Vuorinen et al., 2011; Wang et al., 2021). This proves that, even though it is a quite slow and inefficient alternative route for systemic infection, viruses may invade stems and upper non-inoculated leaves by a cell-to-cell movement through epidermal and mesophyll cells of infected young leaves.

In summary, here, we define ALKBH9B as a new possible factor involved in long-distance transport *via* phloem and we contribute to expanding the knowledge of m^6^A-dependent modulation of plant viral infections. Furthermore, the lack of any evident developmental phenotype in *alkbh9b* mutant plants make ALKBH9B homologs interesting potential targets to develop partial resistances against AMV in important economically crops such as alfalfa or soybean.

## Data Availability Statement

The raw data supporting the conclusions of this article will be made available by the authors, without undue reservation.

## Author Contributions

MM-P, VP, and FA conceived the project, designed the experiments, and wrote the manuscript. CG-M conducted the *in situ* hybridization experiments with the assistance from MM-P. RN and JM generated and characterized *alkbh9a, alkbh9c*, and all possible *alkbh9* double and triple mutant plant combinations. MM-P conducted the rest of the experiments with assistance from LA-M. All authors analyzed and discussed the results, and corrected the manuscript.

## Funding

This research was funded by the Spanish Agencia Estatal de Investigación (AEI), grant numbers PID2020-115571RB-I00 to VP, and PGC2018-093445-B-I00 to JLM. MM-P was recipient of Predoctoral Contract FPI-2015-072406 from the Subprograma Formación de Personal Investigador–Ministerio de Economía y Competitividad (FPI-MINECO). LA-M was recipient of a Predoctoral contract from the Ministerio de Ciencia, Tecnología y Telecomunicaciones from Costa Rica (MICITT-PINN-CON-624-2019). RN was recipient of the GRISOLIAP/2016/131 Predoctoral Contract from the Generalitat Valenciana.

## Conflict of Interest

The authors declare that the research was conducted in the absence of any commercial or financial relationships that could be construed as a potential conflict of interest.

## Publisher’s Note

All claims expressed in this article are solely those of the authors and do not necessarily represent those of their affiliated organizations, or those of the publisher, the editors and the reviewers. Any product that may be evaluated in this article, or claim that may be made by its manufacturer, is not guaranteed or endorsed by the publisher.

## Footnotes

^1^ https://www.arabidopsis.org/
