# Supplementary material for: The m6A RNA Demethylase ALKBH9B Plays a Critical Role for Vascular Movement of Alfalfa Mosaic Virus in Arabidopsis
Source: Front Microbiol. 2021 Oct 4;12:745576. doi: 10.3389/fmicb.2021.745576 (PMC8521051; doi:10.3389/fmicb.2021.745576)
Supplement: Supplementary file 1 [file Table_1.DOCX]

Supplementary Material

**Supplementary Figure Legends**

**Supplementary Figure 1.** Dot-blot hybridization of non-inoculated aerial tissue from WT and *alkbh9b* plants. Two leaves were mechanically inoculated with virus (AMV) or buffer (MOCK) as negative controls. Inoculated leaves were removed at 20 dpi and total RNA from each whole plant was extracted and applied onto nylon membrane to detect the presence of the virus.

**Supplementary Figure 2.** *In situ* hybridization images of the SAM from several AMV-inoculated WT and *alkbh9b* plants. MOCK-inoculated WT plants are included as negative control at 8 dpi. A digoxigenin-labelled probe to detect AMV RNA3 and sgRNA 4 was used to detect the presence of the virus. Scale bars correspond to 100 µm.

**Supplementary Figure 3.** Northern blot analysis to determine AMV virion stability. Upper panel shows accumulation levels of vRNAs extracted from virions purified from three WT and *alkbh9b* plants at 0, 30 and 60 min after the RNase sensitivity assay treatment (see main text). Positions of the AMV RNAs are indicated on the left. Lower panel shows ethidium bromide staining of total RNAs from the same samples.

**Supplementary Figure 4.** *In situ* hybridization images of transversal sections of two AMV-inoculated leaves from WT and *alkbh9b* plants at 4 and 7 dpi. A digoxigenin labelled probe to detect AMV RNA3 and sgRNA 4 was used to detect the presence of the virus. Scale bars correspond to 200 µm.
