# Supplementary material for: The m6A RNA Demethylase ALKBH9B Plays a Critical Role for Vascular Movement of Alfalfa Mosaic Virus in Arabidopsis
Source: Front Microbiol. 2021 Oct 4;12:745576. doi: 10.3389/fmicb.2021.745576 (PMC8521051; doi:10.3389/fmicb.2021.745576)
Supplement: Supplementary file 2 [file Table_2.DOCX]

Supplementary Material

| **Supplementary Table 1.** Primer sets used in this work | | | |
| --- | --- | --- | --- |
| Purpose | Oligonucleotide name(s) | Oligonucleotide sequences (5′ → 3′) | |
|  |  | Forward primer (F or D) | Reverse primer (R) |
| Genotyping | SALK_204823C_F/R | TGACGAATCATGGCATAGTAGC | ACATAAAACATTTGCAAGCGC |
|  | SALK_015591_F/R | CGAGTTCGATGAAGACTCCAG | ATCCTGTTGAATAGAACCGGG |
|  | SALK_021775_F/R | GGGCTGATATGGGAGAAGAAG | GGCAGGTACACAATGCTTAGC |
|  | LbB1.3^a^ |  | ATTTTGCCGATTTCGGAAC |
| RT-PCR | qALKBH9a_F/R | TTCTTTTATCCGGCAGTCGG | ATGGTCAGAGGAGCCACAAG |
|  | qALKBH9b_R^b^ |  | CTACTAGGACGTGGCATTCCT |
|  | qALKBH9c_F/R | CTCGCCAAGTATCAATCATCC | TGTCGGAGCTAGTAACCTTGG |
|  | OTC3D/OTCR^c^ | TCCTTGCCAAATCATGGCCG | GCATGCATGCGATTCTCCGC |

^a,c^Sequences taken from ^a^(Alonso et al., 2003) and ^c^(Quesada et al., 1999). ^b^We also used the SALK_015591_F oligonucleotide to RT-PCR amplify *ALKBH9B* transcripts.

**Supplementary References**

Alonso, J. M., Stepanova, A. N., Leisse, T. J., Kim, C. J., Chen, H., Shinn, P., et al. (2003). Genome-wide insertional mutagenesis of Arabidopsis thaliana. *Science (80-. ).* 301, 653–657. doi:10.1126/science.1086391.

Quesada, V., Ponce, M. R., and Micol, J. L. (1999). OTC and AUL1, two convergent and overlapping genes in the nuclear genome of Arabidopsis thaliana. *FEBS Lett.* 461, 101–106. doi:10.1016/S0014-5793(99)01426-X.
