# Supplementary figures and images for: The m6A RNA Demethylase ALKBH9B Plays a Critical Role for Vascular Movement of Alfalfa Mosaic Virus in Arabidopsis
Source: Front Microbiol. 2021 Oct 4;12:745576. doi: 10.3389/fmicb.2021.745576 (PMC8521051; doi:10.3389/fmicb.2021.745576)

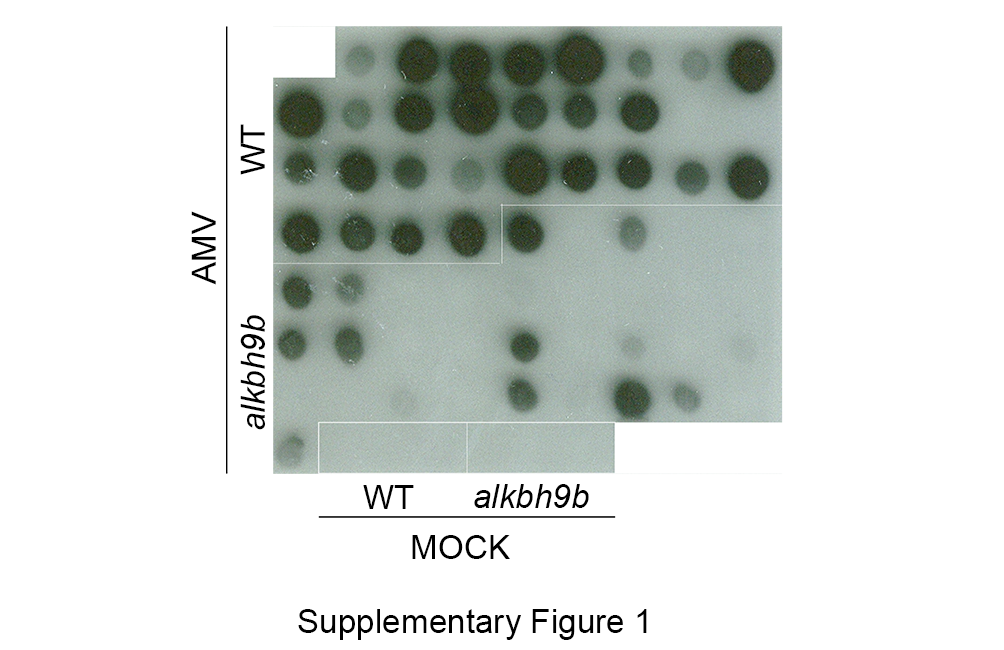

Supplement: Supplementary file 3 [file Image_1.TIF]

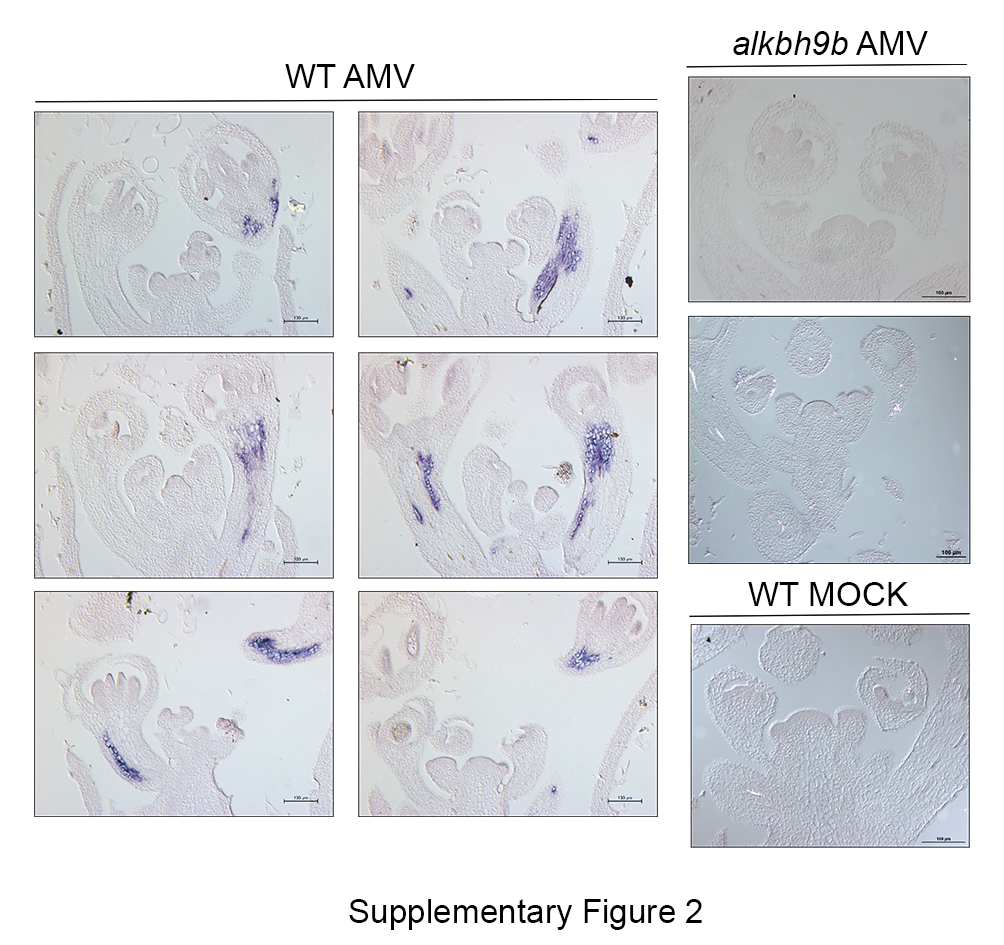

Supplement: Supplementary file 4 [file Image_2.TIF]

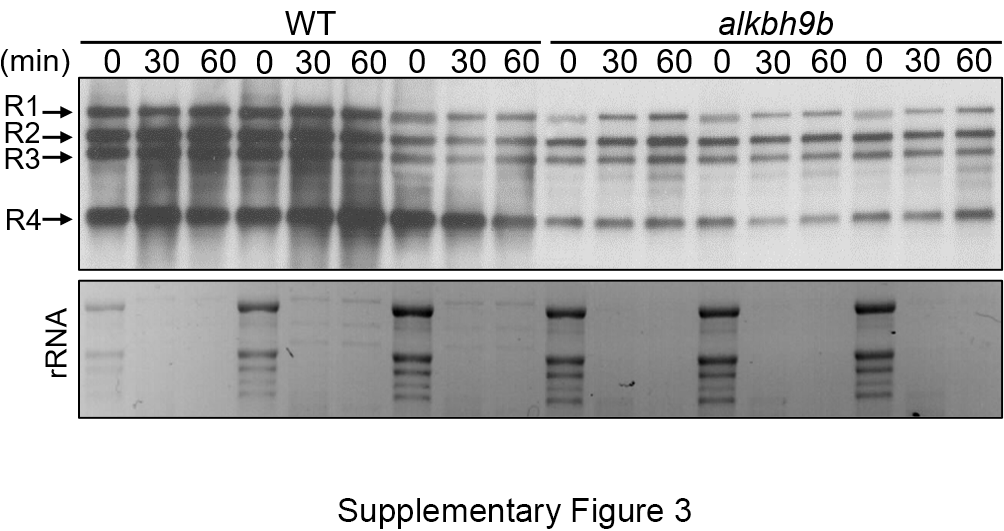

Supplement: Supplementary file 5 [file Image_3.TIF]

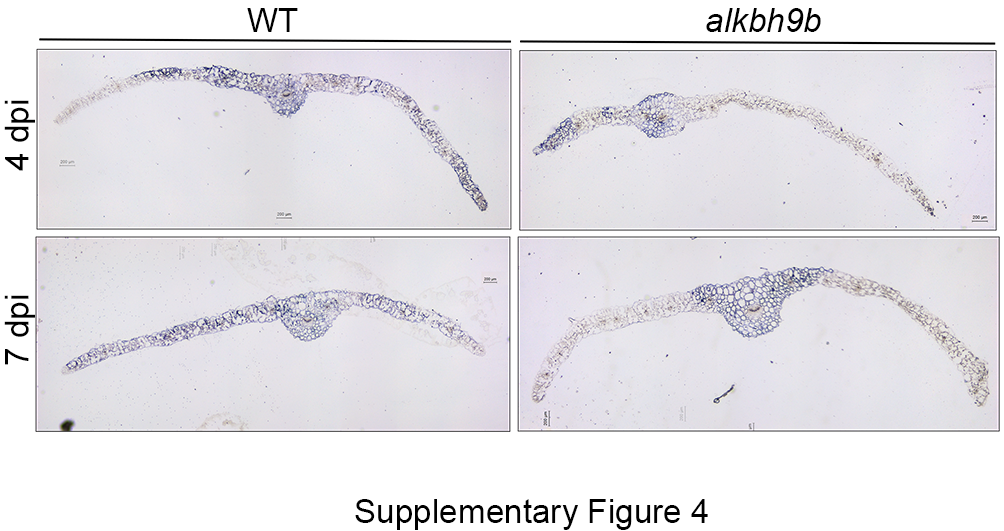

Supplement: Supplementary file 6 [file Image_4.TIF]
